# Pavement 3D Data Denoising Algorithm Based on Cell Meshing Ellipsoid Detection

**DOI:** 10.3390/s21072310

**Published:** 2021-03-25

**Authors:** Chuang Yan, Ya Wei, Yong Xiao, Linbing Wang

**Affiliations:** 1 Department of Civil Engineering, Tsinghua University, Haidian District, Beijing 100084, China; c-yan19@mails.tsinghua.edu.cn (C.Y.); xiaoyong17@mails.tsinghua.edu.cn (Y.X.); 2 Virginia Tech, Blacksburg, VA 24061, USA; wangl@vt.edu

**Keywords:** 3D reconstruction, denoising algorithm, ellipsoid neighborhood, pavement engineering, point cloud data

## Abstract

As a new measuring technique, laser 3D scanning technique has advantages of rapidity, safety, and accuracy. However, the measured result of laser scanning always contains some noise points due to the measuring principle and the scanning environment. These noise points will result in the precision loss during the 3D reconstruction. The commonly used denoising algorithms ignore the strong planarity feature of the pavement, and thus might mistakenly eliminate ground points. This study proposes an ellipsoid detection algorithm to emphasize the planarity feature of the pavement during the 3D scanned data denoising process. By counting neighbors within the ellipsoid neighborhood of each point, the threshold of each point can be calculated to distinguish if it is the ground point or the noise point. Meanwhile, to narrow down the detection space and to reduce the processing time, the proposed algorithm divides the cloud point into cells. The result proves that this denoising algorithm can identify and eliminate the scattered noise points and the foreign body noise points very well, providing precise data for later 3D reconstruction of the scanned pavement.

## 1. Introduction

As a new non-contact measurement method, the 3D laser scanning technique obtains the coordinates of the points in a scanned object by using the triangle measurement based on the distance from the scanning device to the scanned points and the angle of the reflected light [1,2,3]. Compared to the traditional measuring technique, such as manual measurement or visual inspection, laser scanning technique shows the advantages of safe operation, accurate measurement, and high efficiency, which becomes the promising technique for pavement detection [4]. However, like other measuring methods, the result of laser scanning inevitably contains noise points that do not belong to the pavement, which adversely affects the 3D reconstruction of pavement as well as the defect identification in the later stage. The causes of noise points are various [5,6], including the environmental interference (such as dust in the air), the foreign body, the sharp changes of laser incidence angle, etc.

Cao [7] classified noise points into three categories: scattered noise points deviating from the target object, less dense noise points deviating from the target object, and noise points mixed with the target object. For pavement point cloud, noise points can also be divided into these three categories. Because of the strong planarity feature of pavement, noise points are all above this pavement plane. These noise points need to be identified and eliminated by using the specific algorithms. The process of identification and elimination of noise points is called denoising process [8]. However, limited by the scanning principle and the equipment of the 3D laser scanning, the point cloud data includes only the coordinates and the RGB values of each point, which makes the denoising process difficult because the topological connection between points are not included [9].

The traditional denoising algorithms include robust filtering [10,11], median filtering [12], mean filtering [13], least squares algorithms [14], and so on. Based on the traditional algorithms, some new denoising algorithms have been proposed to eliminate noise points more accurately and quickly. Liu et al. [15] proposed a robust denoising algorithm based on the kernel density estimation clustering algorithm to eliminate the surface noise points of the general 3D point cloud and to achieve the efficient and fast denoising. Mattei et al. [16] proposed the motion robust principle component analysis (MRPCA) to denoise 3D point cloud and at the same time retain the sharp characteristics of the point cloud data by dividing the denoising process into the local and the global parts. Rakotosaona et al. [17] proposed a deep-learning-based approach for estimating the local 3D shape properties of point clouds, which shows good robustness and effectiveness when dealing with the surface noise points. The above algorithms are aimed at denoising the general 3D point cloud data, of which some algorithms were applied to denoise the pavement point cloud data [18]. However, the contributions of each coordinates of the scanned points of pavement are equally treated by these algorithms during the denoising process, which ignores the strong planarity feature of the pavement point cloud data. It is possible that these algorithms even treat the foreign body (such as kerbs or stones on the pavement) as part of the pavement or cause the defects over-smoothened, which results in the precision loss of the 3D reconstruction.

In view of the denoising problem caused by applying the traditional algorithms, the algorithms aimed at denoising the pavement 3D point cloud data has been proposed. Wang [19] proposed a multi-level denoising model to ensure the accuracy of the crack identification in the later stage by adding some adaptive supplements to improve the feasibility of algorithms. Vosselman [20] proposed the maximum local slope filtering algorithm, which mainly utilizes the slope of different cloud blocks to identify noise points. Petzold et al. [21] proposed the moving window least squares fitting algorithm, which fits the surface according to the lowest point of each window and eliminates the points with distance to the surface exceeding the threshold. Liu et al. [22] proposed a denoising algorithm for pavement point cloud data by a vehicle-borne scanner. The noise points are identified and eliminated through the cluster analysis. Hu et al. [23] proposed a denoising algorithm based on the deep learning, which distinguishes the ground points from the non-ground points by deep convolution neural network. Although the above algorithms took the strong planarity feature of the pavement into account, the parameters in their algorithms need to be manually adjusted many times for an ideal denoising result, which increases the program complexity. Lin et al. [24] proposed a segmentation based-filtering algorithm (SBF), which can identify and eliminate the pavement noise points by point cloud segmentation, multi-echo analysis, and iterative judgment. Qiu et al. [25] utilized the neighborhood to eliminate the scattered noise points. Liu et al. [22] and Qiu et al. [25] mainly aimed at denoising vehicle scanned point cloud. The density and accuracy of the scanned points are greatly affected by the vehicle speed and the road condition, which makes these algorithms not suitable for denoising the point cloud data obtained under high accuracy measurement.

To highlight the contribution of the horizontal coordinates of the pavement points, this study utilizes the characteristic of the oblate ellipsoid of which the equatorial radius is larger than the polar radius to denoise the data points of pavement with strong planarity feature. Moreover, the point cloud is divided into cells before the ellipsoid detection to narrow down the space of the ellipsoid detection and to save computing time. The rest of this paper is organized as follows. Section 2 introduces the theory of the denoising algorithm proposed in this study. Section 3 and Section 4 are the numerical analysis of Section 2, including the meshing stage and the ellipsoid detection, respectively. The algorithm verifications by using both the simulated and the scanned point cloud are presented in Section 5. Finally, Section 6 concludes the paper.

## 2. Theory of Denoising Algorithm

**Figure 1:**
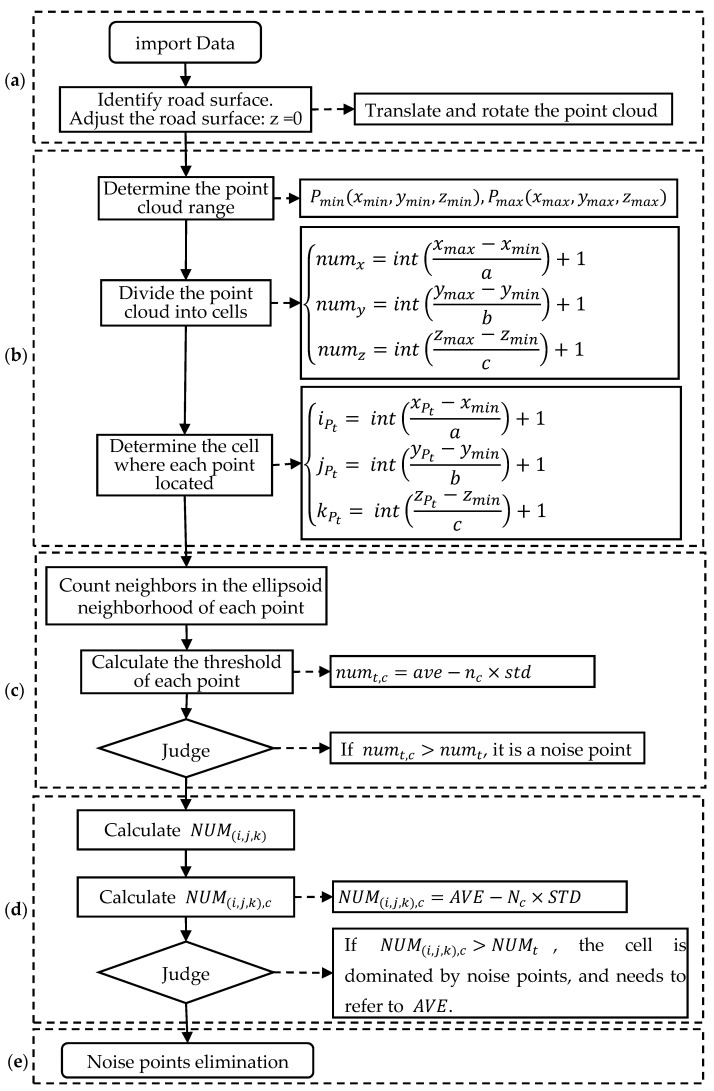
The flow chart of pavement point cloud denoising algorithm based on cell meshing ellipsoid detection: (**a**) Preparation stage; (**b**) cell meshing the point cloud and pre-denoising; (**c**) Denoising by ellipsoid detection; (**d**) Comparison between cells; (**e**) Noise points elimination.

A denoising algorithm for pavement points cloud data is proposed based on the cell meshing ellipsoid detection in this study (Figure 1). Considering the planarity feature of pavement surface, this denoising algorithm utilizes the ellipsoid detection to highlight the contribution of the horizontal coordinates to the threshold of the denoising. In detail, an ellipsoid neighborhood for each point is constructed, and the number of the neighbors in this neighborhood is counted. Based on the normal distribution principle, each point has its threshold which distinguishes if this point is a noise point or not. A point will be identified as a noise point if the number of neighbors in its ellipsoid neighborhood is less than the threshold.

**Figure 2:**
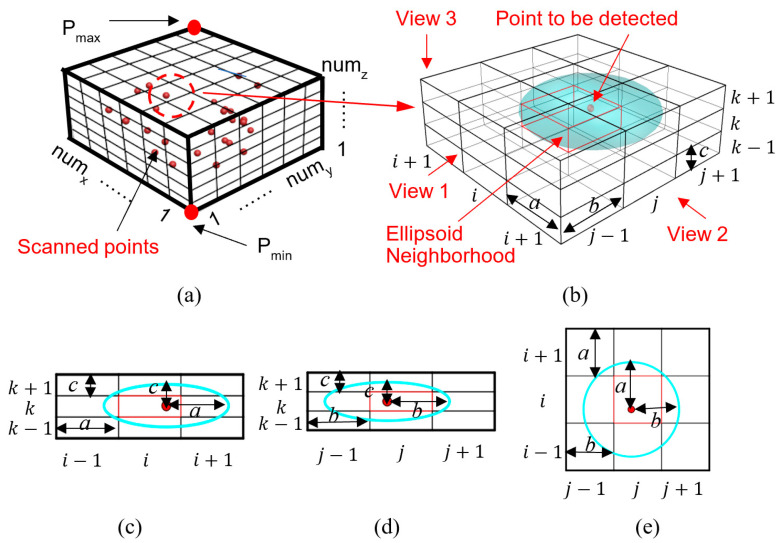
(**a**) The schematic diagram of cell meshing point cloud, (**b**) the surrounding cells of the point to be detected and (**c**) view 1, (**d**) view 2 and (**e**) view 3 of the (**b**), respectively.

Moreover, this study divides the entire point cloud into cells before denoising to reduce the spatial range of the ellipsoid detection for higher denoising efficiency. The point to be detected is the center point of its ellipsoid neighborhood, of which the neighbors are counted. Because the width and the length of the gird equal to the equatorial radius of the ellipsoid and the height of the gird equals to the polar radius of the ellipsoid, the ellipsoid neighborhood fits in 27 cells (3 × 3 × 3) (Figure 2). Thus, the ellipsoid detection is conducted in both the cell where the point is located and its surrounding cells. This operation effectively reduces the computing time due to the narrow down of the detection space. The detailed process is discussed as follows (as shown in Figure 1).

(a) Preparation stage: To stress the strong planarity characteristic of pavement in the denoising process, the pavement plane is firstly identified by utilizing the visualization software, such as Geomagic Studio 2012. The identified pavement plane is then represented by a horizontal plane (the *x*–*y* plane with *z* = 0), and the normal direction of the pavement plane denotes the height direction (*z*-axis direction). 

(b) Cell meshing stage: As shown in Figure 2a, the point with the smallest *x*, *y*, *z* coordinates among all points are denoted as $P_{min}(x_{min},y_{min},z_{min})$, and the point with the largest *x*, *y*, *z* coordinates among all points are denoted as $P_{max}(x_{max},y_{max},z_{max})$. All points are distributed in the cuboid space with the diagonal vertices of $P_{min\;}$ and $P_{max}$, and the cuboid edges are along the *x*-axis, *y*-axis, or *z*-axis direction. The point cloud is then divided into cells. The length and width of the cell are equal to the equatorial radius of the ellipsoid, and the height of the cell is equal to the polar radius of the ellipsoid. The known pavement plane is at *z* = 0, and it is assumed that the ground points is distributed within an extremely limited range along the height direction. Therefore, the points will be considered as noise points and to be eliminated if they locate in cells far from the pavement plane. 

(c) Ellipsoid detection stage: The ellipsoid detection is done for each point in the cloud to detect if one point is a noise point. The denoising critical value of one point should be first determined to judge whether this point is a noise point, and the detailed process is as follows. The number of neighbors in the ellipsoid neighborhood of $P_{t}$ ($P_{t}$ is the *t*th point in the point cloud) is denoted as $num_{t}$. Its neighbors are denoted as $P_{t,i}\left(i=1,2,3\ldots \right)$, and the number of neighbors in the ellipsoid neighborhood of $P_{t,i}$ is denoted as $num_{t,i}$. The denoising critical value of $P_{t}$ is denoted as $num_{t,c}$, which is calculated according to the average value (*ave*) and the standard variance (*std*) of the array $num_{t,i}\left(i=1,2,3\ldots \right)$, as shown in Equation (1). If $num_{t}\;$<$\;num_{t,c}$, $P_{t}$ will be identified as a noise point. Parameter $n_{c}$ is used to control the denoising degree. If the array $num_{t,i}$ obeys the normal distribution, the probability that $P_{t}$ is a noise point is 99.87% when $n_{c}$ = 3 and $num_{t}$ < $num_{t,c}$. When the scanned data is sufficient, $n_{c}$ can be reduced to increase the denoising critical value ($num_{t,c}$) to eliminate more potential noise points but possibly eliminate some ground points as well.

(1) $$num_{t,c}=ave(num_{t,i})-n_{c}\times std(num_{t,i})$$

(d) Comparing stage: The comparison of the types of majority points in different cells is done to eliminate the remained noise points after stage (c). This stage applies to the situation that most points in one cell are noise points, and thus the critical value calculated in stage (c) is small. This small critical value may not be able to judge whether a point is a noise point. Therefore, to identify the cells containing mainly noise points, the comparison of the average number of neighbors in the ellipsoid neighborhood of each point from different cells is carried out. The identification principle is illustrated below. 

As shown in Figure 2a, *i*, *j*, and *k* are the cell sequence numbers along *x*-axis, *y*-axis, and *z*-axis direction, respectively. For cell *G*(*i*, *j*, *k*), the mean value of all internal points’ number of neighbors is taken as the eigenvalue of the cell, which is denoted as $NUM_{\left(i,j,k\right)}$. The critical value $NUM_{\left(i,j,k\right),c}$ of cell *G*(*i*, *j*, *k*) is calculated according to the average value (*AVE*) and the standard variance (*STD*) of the eigenvalues of the surrounding cells (i.e., *G*(*i*±△*i*, *j*±△*j*, *k*±△*k*), △*i* = −1, 0, +1; △*j* = −1, 0, +1; △*k* = −1, 0, +1, and they are not 0 at the same time), as shown in Equation (2). Parameter $N_{c}$ is used to control the denoising degree. Similar to the determination of $n_{c}$ in Equation (1)**,** if the array $NUM_{\left(i\pm \Delta i,j\pm \Delta j,k\pm \Delta k\right)}$ obeys the normal distribution, the probability that *G(i, j, k)* is a cell in which most points are noise points is 99.87% when $N_{c}$ = 3 and $NUM_{\left(i,j,k\right)}$ < $NUM_{\left(i,j,k\right),c}$. Therefore, the critical value of each point inside the cell *G*(*i*, *j*, *k*) will all be increased from $num_{t,c}$ (calculated in stage c) to $NUM_{\left(i,j,k\right),c}$ to avoid the occurrence of a small critical value determined by Equation (1).

(2) $$NUM_{\left(i,j,k\right),c}=AVE\left(NUM_{\left(i\pm \Delta i,j\pm \Delta j,k\pm \Delta k\right)}\right)-N_{c}\times STD\left(NUM_{\left(i\pm \Delta i,j\pm \Delta j,k\pm \Delta k\right)}\right)$$

(e) Ending stage: The denoising result can be obtained after noise points have been identified and eliminated, and then the 3D reconstruction can be done. The feasibility of the denoising algorithm in terms of theoretical analysis will be discussed below.

## 3. Discussion of Cell Meshing Stage

### 3.1. Significance and Construction of Cells

This study proposes the approach of ellipsoid detection to distinguish noise points, and the neighbors in the ellipsoid neighborhood of each point need to be obtained first. However, if all points are checked to see if they are in the ellipsoid neighborhood, the program complexity will be greatly increased, given the huge amount of points. Therefore, the ellipsoid detection should be carried out in the partial space of the 3D point cloud by dividing the point cloud into cells, and the ellipsoid detection is carried out in the partial space composed of cells, as shown in Figure 2b. 

For each point, the ellipsoid detection is carried out within both the cell that the point belongs to and its surrounding cells. At most 27 surrounding cells are involved during the ellipsoid detection, the inner cell has 27 surrounding cells, and the cells at the circumferences has less than 27 surrounding cells. As illustrated in Figure 2c–e, the point for the ellipsoid detection is represented by the red point, and its ellipsoid neighborhood locates at its surrounding cells. To detect the points belonging to the boundary of the target area, the target area is enlarged by a cell size in the horizontal direction when conducting the ellipsoid detection. The points in the added cells serve as the reference to determine the critical value of the boundary points. 

The number of cells along the x-axis, y-axis, and z-axis direction is denoted as $num_{x},$ $num_{y}$ and $num_{z}$, respectively. If each cell has n points, there are total $num_{x}\times num_{y}\times num_{z}\times n$ points in the point cloud. If each point in the point cloud needs to be detected, total $num_{x}\times num_{y}\times num_{z}\times n$ detections are required. After the point cloud is divided into cells, only the points in the surrounding 27 cells are checked, and only 27 × n detections are required, and the denoising time will be reduced by $num_{x}\times num_{y}\times num_{z}/27$ times compared to that without cell construction. Therefore, the cell meshing operation can greatly reduce the computing time.

The detailed cell meshing process is as follows:

(a) The coordinates of $P_{min}(x_{min},y_{min},z_{min})$ and $P_{max}(x_{max},y_{max},z_{max})$ are determined.

(b) The number of cells along *x*-axis, *y*-axis or *z*-axis direction is calculated by Equation (3), and the output of int(input) is the integer part of the input.

(3) $$\{\begin{array}{c} num_{x}=int\left(\frac{x_{max}-x_{min}}{a}\right)+1\\ num_{y}=int\left(\frac{y_{max}-y_{min}}{b}\right)+1\\ num_{z}=int\left(\frac{z_{max}-z_{min}}{c}\right)+1 \end{array}$$

(c) Assuming $P_{t}$ is the *t*th point, its *x*, *y*, and *z* coordinates are $x_{P_{t}}$, $y_{P_{t}}$ and $z_{P_{t}}$, respectively. The cell where $P_{t}$. is located is denoted as $G\left(i_{P_{t}},\;j_{P_{t}},\;k_{P_{t}}\right)$, and $i_{P_{t}}$, $j_{P_{t}}$ and $k_{P_{t}}$ are the sequence numbers along the *x*-axis, *y*-axis, and *z*-axis direction, respectively. $i_{P_{t}}$, $j_{P_{t}}$ and $k_{P_{t}}$ are determined by Equation (4). Particularly, $i_{P_{min}}$, $j_{P_{min}}$ and $k_{P_{min}}$ are 1, and $i_{P_{max}}$, $j_{P_{max}}$ and $k_{P_{max}}$ are $num_{x}$, $num_{y}$ and $num_{z}$, respectively.

(4) $$\{\begin{array}{c} i_{P_{t}}=\;int\left(\frac{x_{P_{t}}-x_{min}}{a}\right)+1\\ j_{P_{t}}=\;int\left(\frac{y_{P_{t}}-y_{min}}{b}\right)+1\\ k_{P_{t}}=\;int\left(\frac{z_{P_{t}}-z_{min}}{c}\right)+1 \end{array}\left(1\leq t\leq \mathrm{the}\;\mathrm{number}\;\mathrm{of}\;\mathrm{points}\right)$$

After the above cell meshing process, the cell where each point is located is determined. To further simply the ellipsoid detection and reduce the computing time, pre-denoising can be done to eliminate some obvious noise points, and the details are discussed in the next section. 

### 3.2. Pre-Denoising Point Cloud

Some obvious noise points can be eliminated in advance by utilizing the relationship between the cells. The ground points approximately make up a plane located at the bottom of the point cloud; therefore, these ground points are distributed at the bottom cells. By utilizing this feature, we can eliminate some noise points before the ellipsoid detection.

The detailed pre-denoising process is as follows:

(a) For *i* from 1 to *num_x_* and for *j* from 1 to *num_y_*, points are detected from the bottom cell to the top cell (for *k* from 1 to *num_z_*). The value of *k* when the point is detected for the first time is denoted as $k_{exist}^{i,\;j}$. 

(b) Because the z-coordinates of the ground points vary in a small range, it can be inferred that the points in the cell that are over a critical height from *G*$\left(i,j,k_{exist}^{i,\;j}\right)$ are all noise points. This critical height is denoted as $h_{c}$, which is a positive integer and determined from the z-coordinates change of the ground points and the size of the cell. That is all points in the $G\left(i,j,k_{exist}^{i,\;j}+h\right)\left(h>h_{c}\right)$ are treated as noise points.

(c) $k_{exist}^{i,\;j}$ is compared to that of the surrounding cells ($k_{exist}^{i\pm \Delta i,\;j\pm \Delta j}$) (Δi = −1, 0, +1. Δj = −1, 0, +1. Δi and Δj cannot equal to 0 at the same time). If the difference ($k_{exist}^{i,\;j}-k_{exist}^{i\pm \Delta i,\;j\pm \Delta j}$) is larger than $h_{c}$, it is considered that the points in $G\left(i,j,k_{exist}^{i,\;j}\right)$ are much higher than the points in $G\left(i\pm \Delta i,j\pm \Delta j,k_{exist}^{i\pm \Delta i,\;j\pm \Delta j}\right)$, and thus, all points in $G\left(i,j,k\right)\;\left(1\leq k\leq num_{z}\right)$ need to be eliminated. After this operation, the point cloud is pre-denoised.

## 4. Analysis of Ellipsoid Detection

The ellipsoid detection method is proposed in this study to distinguish both the scattered noise points and the foreign body noise points from the ground points, which highlights the contribution of the horizontal coordinates of the scanned points to the denoising threshold. The influencing factors, including the ellipsoid shape, the distance from the point to the pavement, and the included angle between the foreign body and the pavement, will be analyzed to find out the appropriate ellipsoid shape for denoising.

Before the detailed analysis, the following assumptions are made for simplifying the numerical analysis:

(a) Within a small space, the ground points or the foreign body noise points are evenly distributed. The number of the neighbors in one point’s ellipsoid neighborhood is proportional to the intersection area between the pavement plane and the ellipsoid neighborhood or the intersection area between the foreign body plane and the ellipsoid neighborhood.

(b) The z-coordinates variation of the ground points is limited in a small range and can form a plane within a small area of 0.40 m × 0.40 m in this study, as expressed in Equation (5).

(5) z=constant value

(c) By assuming a=b, the influence of *x* and *y* coordinates on the denoising results is unified. *a* and *b* are the equatorial radiuses of the ellipsoid neighborhood.

(d) To simplify the later calculation, the polar radius of the ellipsoid neighborhood c is fixed as 1 mm. Because the equatorial radius of the oblate ellipsoid is larger than the polar radius of the oblate ellipsoid, it has a=b and a/c>1.

(e) The vertical distance (or height) from the point to be detected to the pavement plane is denoted as d. Since *c* = 1 mm, when 0<d≤c=1 mm, there exists ground points in the ellipsoid neighborhood of the point. When d>c=1 mm, there is no ground points in the ellipsoid neighborhood of that point.

**Figure 3:**
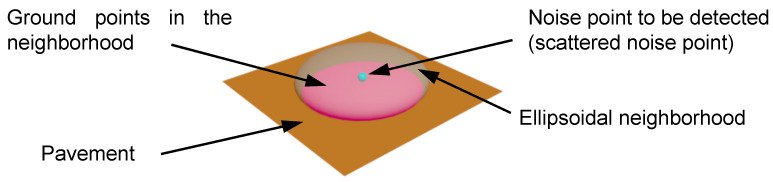
Ground points in the Ellipsoidal neighborhood of the scattered noise point.

Figure 3 shows the obtained point cloud consisting of ground points, the scattered noise points, and the foreign body noise points. To verify the effectiveness of the ellipsoid detection in distinguishing different types of noise points, the detailed calculation is made to obtain the different intersection areas in the ellipsoid neighborhood of the scattered noise point, the foreign body noise point, and the ground point. The denoising ability of the ellipsoid detection can be quantified by the intersection area.

### 4.1. Sensitivity Analysis for Detecting Scattered Points

Since the density of the scattered points is far less than that of the ground points, the scattered points cannot form a plane. Therefore, the intersection area between the pavement plane and the ellipsoid neighborhood of the scattered points is actually part of the pavement plane, as the pink area shown in Figure 3. To discuss the denoising ability of the ellipsoid detection for the scattered points, this intersection area is numerically calculated and compared with that of the ground point. 

To detect if a scattered points $P_{scatter}$ is a noise point or not, the coordinates of this point are set as (0,0,0) for the convenience of calculation. Its height from the pavement plane is denoted as d. The equation of the ellipsoid neighborhood of $P_{scatter}$ is expressed as:

(6) $$\frac{x^{2}}{a^{2}}+\frac{y^{2}}{b^{2}}+z^{2}=1\;\left(a=b\right)$$

By combining Equations (5) and (6), the equation of the intersection area between the ellipsoid neighborhood of $P_{scatter}$ and the pavement plane is shown as Equation (7), where the constant value in the Equation (5) is −d.

(7) $$\frac{x^{2}}{a^{2}\left(1-d^{2}\right)}+\frac{y^{2}}{b^{2}\left(1-d^{2}\right)}=1\;\left(a=b\right)$$

The denoising ability of the ellipsoid detection can be quantified by the intersection area. The smaller the intersection area, the more likely the point to be detected is a noise point. However, the intersection areas of the ellipsoid neighborhood of the scattered point and that of the ground point are different. For example, the intersection area between the pavement plane and the ellipsoid neighborhood of the scattered points is $a^{2}\left(1-d^{2}\right)\pi$ mm^2^ (the pink zone shown in Figure 3) by Equation (7). For ground point, its intersection area between the pavement plane and the ellipsoid neighborhood of this point is $\left(a^{2}\pi \right)$ mm^2^. Therefore, a normalization of the intersection area aiming to eliminate the impact of the ellipsoid shape should be done to conveniently compare and analyze the denoising sensibility of the ellipsoid detection.

The normalization process is conducted through dividing the intersection area of the ellipsoid neighborhood of a scattered point by the intersection area of the ellipsoid neighborhood of a ground point. The normalized result equals to $\left(1-d^{2}\right)$, which is denoted as *S_scatter_*, as shown in Equation (8).

(8) $$S_{scatter}=1-d^{2}$$

It is specified in this study that a scattered point will be easily identified as a noise point if its $S_{scatter}$ is less than 0.5. From Equation (8), *S_scatter_* < 0.5 if *d* > 0.71 mm; that is, the number of neighbors in the neighborhood of the scattered points is less than half of that in the ground point’s neighborhood, and thus the point is detected as a scattered noise point.

The influence of *c* (the ellipsoid polar radius, along *z*-axis direction) is revealed that the points within the distance of 0–0.71*c* from the pavement plane can be accepted as the ground points. This is because *c* equals to 1 mm by assumption to simplify the calculation in the above analysis, and therefore, by adjusting the value of *c*, the accepted distance from the pavement plane within which the scattered points can be regarded as the ground points can be controlled to a relatively small value to eliminate more scattered noise points and to maintain the ground points.

### 4.2. Sensitivity Analysis for Detecting Foreign Body Points

Affected by the scanning environment, foreign body such as stones, kerbs, and branches on the pavement may be scanned, and these scanned noise points should also be eliminated. Unlike the scattered points, the foreign body points are dense and mixed with the ground points. Therefore, the number of neighbors in the ellipsoid neighborhood of the ground points and that of the foreign body points can be represented by their intersection area, respectively.

#### 4.2.1. Constructing Ellipsoid Neighborhood for Foreign Body Points

**Figure 4:**
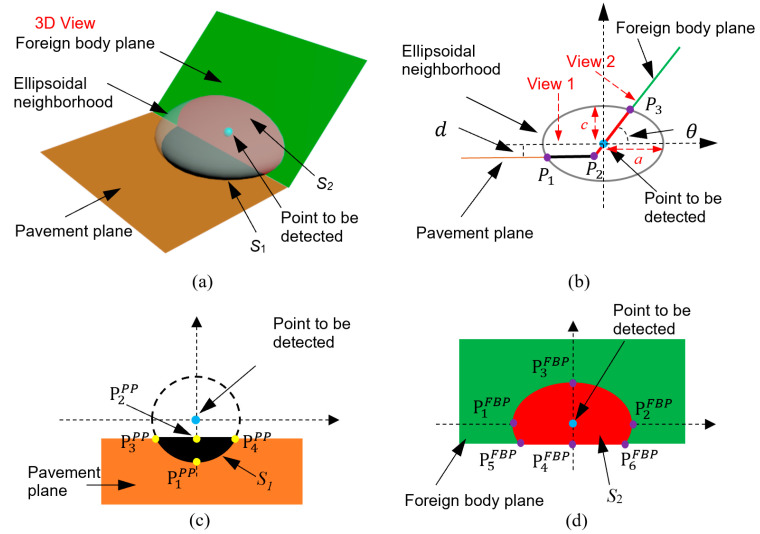
Schematic diagram of the ellipsoid detection of point cloud containing the foreign body, (**a**) 3D view, (**b**) side view of (**a**), (**c**) view 1 of (**b**) and (**d**) view 2 of (**b**).

As shown in Figure 4a, the local foreign body surface can be treated as a plane when the ellipsoid is small. The intersection area between the ellipsoid and the pavement plane is denoted as $S_{1}$, and the intersection area between the ellipsoid and the foreign body is denoted as $S_{2}$. The point to be detected whether a foreign body noise points or a ground point is located in the center of its ellipsoid neighborhood. The distance from the point to the pavement is *d* (Figure 4b). The included angle between the foreign body and the pavement is denoted as (180°−θ). tanθ is denoted as *k* to simplify calculation. Figure 4b is the side view of Figure 4a, and the location of the point to be detected is treated as the origin of the coordinate system. The equations of ellipsoid, the pavement plane, and the foreign body plane shown in Figure 4b are expressed as Equation (9), Equation (10) and Equation (11), respectively:

(9) $$\frac{x^{2}}{a^{2}}+y^{2}=1\;(\mathrm{for}\;\mathrm{ellipsoid})$$

(10) y=−d (for pavement plane)

(11) y=kx  (k=tanθ, for foreign body plane)

As shown in Figure 4b, $P_{1}$ is the intersection point of the ellipsoid and the pavement plane. By combining Equations (9) and (10), the coordinates of $P_{1}$ are calculated as $\left(-a\sqrt{1-d^{2}},-d\right)$. $P_{2}$ is the intersection point of the pavement plane and the foreign body plane. The coordinates of $P_{2}$ are obtained as $\left(-\frac{d}{tan\theta },-d\right)$ by combining Equations (10) and (11). $P_{3}$ is the intersection point of the ellipsoid and the foreign body plane, with coordinates of $P_{3}$ $\left(\frac{a}{\sqrt{1+{\left(ak\right)}^{2}}},\frac{ak}{\sqrt{1+{\left(ak\right)}^{2}}}\right)$ obtained by combining Equations (9) and (11). The intersection areas of $S_{1}$ and $S_{2}$ can be calculated from the coordinates of $P_{1}$, $P_{2}$ and $P_{3}$, which are used to further calculate the total intersection area for sensitivity analysis of detecting foreign body noise point.

#### 4.2.2. Calculation of $S_{1}$

$S_{1}$ is the intersection area between the pavement plane and the ellipsoid neighborhood of the foreign body point to be detected. The sketch of $S_{1}$ on the pavement plane is shown in Figure 4c. Critical points such as $P_{1}^{PP}$, $P_{2}^{PP}$, $P_{3}^{PP}$, $P_{4}^{PP}$ and $P_{5}^{PP}$ are labelled on Figure 4c, and their coordinates are calculated as follows.

(a) $P_{1}^{PP}$ in Figure 4c is corresponding to $P_{1}$ in Figure 4b. The coordinates of $P_{1}^{PP}$ are $\left(0,-a\sqrt{1-d^{2}}\right)$ in Figure 4c.

(b) $\;P_{2}^{PP}$ in Figure 4c is corresponding to $P_{2}$ in Figure 4b. The coordinates of $P_{2}^{PP}$ are $\left(0,-\frac{d}{tan\theta }\right)$ in Figure 4c.

(c) By substituting $y=-\frac{d}{tan\theta }$ into Equation (7) (the equation for calculating the projection of the ellipsoid on the pavement plane), the coordinates of $P_{3}^{PP}$ and $P_{4}^{PP}$ are obtained as $\left(-\sqrt{a^{2}\left(1-d^{2}\right)-\frac{d^{2}}{tan^{2}\theta }},-\frac{d}{tan\theta }\right)$ and $\left(+\sqrt{a^{2}\left(1-d^{2}\right)-\frac{d^{2}}{tan^{2}\theta }},-\frac{d}{tan\theta }\right)$ respectively, in Figure 4c. If $\frac{d}{tan\theta }>\sqrt{a^{2}\left(1-d^{2}\right)}$, it means that the distance from the foreign body point to be detected to the pavement plane is so far that there is no ground point in the ellipsoid neighborhood. In this situation, $P_{3}^{PP}$ and $P_{4}^{PP}$ do not exist and $S_{1}=0$ mm^2^.

By utilizing Equation (7) and the coordinates of $P_{3}^{PP}$ and $P_{4}^{PP}$, $S_{1}$ (mm^2^) can be determined as shown in Equation (12).

(12) $$S_{1}=2\times \int_{0}^{\sqrt{a^{2}\left(1-d^{2}\right)-\frac{d^{2}}{tan^{2}\theta }}}\left(\sqrt{a^{2}\left(1-d^{2}\right)-x^{2}}-\frac{d}{tan\theta }\right)\;dx$$

#### 4.2.3. Calculation of $S_{2}$

$S_{2}$ is the intersection area between the foreign body plane and the ellipsoid neighborhood of the foreign body point to be detected, the sketch of $S_{2}$ on the foreign body plane is shown in Figure 4d. To calculate $S_{2}$, the projection of the foreign body point to be detected on the foreign body plane (FBP) is treated as the origin of the coordinate system in Figure 4d. Critical points such as $P_{1}^{FBP}$, $P_{2}^{FBP}$, $P_{3}^{FBP}$, $P_{4}^{FBP}$, $P_{5}^{FBP}$, and $P_{6}^{FBP}$ are labelled on Figure 4d, and their coordinates are calculated as follows.

(a) Because the red area in Figure 4d is a section of the ellipse with the length of the semi-major axis of *a*, $P_{1}^{FBP}\left(-a,0\right)$ and $P_{2}^{FBP}\left(+a,0\right)$ can be obtained.

(b) $P_{3}^{FBP}$ in Figure 4d is corresponding to $P_{3}$ in Figure 4b. Considering the projection angle (*θ, k = tanθ*), the coordinates of $P_{3}^{FBP}$ are calculated to be $\left(0,\frac{a\sqrt{1+k^{2}}}{\sqrt{1+{\left(ak\right)}^{2}}}\right)$.

(c) $P_{4}^{FBP}$ in Figure 4d is corresponding to $P_{2}$ in Figure 4b. Considering the projection angle (*θ*), the coordinates of $P_{4}^{FBP}$ are calculated to be $\left(0,-\frac{d}{sin\theta }\right)$.

(d) By combining the line equation $y=-\frac{d}{sin\theta }$ and the ellipse equation constructed by $P_{1}^{FBP}$, $P_{2}^{FBP}$ and $P_{3}^{FBP}$, the coordinates of $P_{5}^{FBP}$ and $P_{6}^{FBP}$ are calculated to be $\left(-a\sqrt{1-\frac{d^{2}\left(1+{\left(ak\right)}^{2}\right)}{a^{2}\left(1+k^{2}\right)sin^{2}\theta }},-\frac{d}{sin\theta }\right)$ and $\left(+a\sqrt{1-\frac{d^{2}\left(1+{\left(ak\right)}^{2}\right)}{a^{2}\left(1+k^{2}\right)sin^{2}\theta }},-\frac{d}{sin\theta }\right)$, respectively, in Figure 4d.

Above all, $S_{2}$ (mm^2^) can be determined as shown in Equation (13). If there is no ground point in the ellipsoid, the lower bound of integral in Equation (13) will be replaced by $-\frac{a\sqrt{1+k^{2}}}{\sqrt{1+{\left(ak\right)}^{2}}}$, which means the *S*_2_ area is a complete ellipse.

(13) $$S_{2}=2\times \int_{-\frac{d}{sin\theta }}^{\frac{a\sqrt{1+k^{2}}}{\sqrt{1+{\left(ak\right)}^{2}}}}a\sqrt{1-\frac{1+{\left(ak\right)}^{2}}{a^{2}\left(1+k^{2}\right)}y^{2}}\;dy\;\left(k=tan\theta \right)$$

The total intersection area in the ellipsoid neighborhood of the foreign body point to be detected is $S_{1}$ plus $S_{2}$, which is compared to the intersection area in the ellipsoid neighborhood of the ground point to analyze the denoising ability of the ellipsoid detection. Therefore, the total area is divided by the area of $\pi a^{2}$ mm^2^, and the normalized area (*S_foreign body_*) is determined by Equation (14). It is seen that *S_foreign body_* is influenced by the ellipsoid parameter (a), the included angle (θ), and the distance from the foreign body point to be detected to the pavement plane (*d*).

(14) $$S_{foreign\;body\;}=\left(S_{1}+S_{2}\right)/\left(a^{2}\pi \right)$$

#### 4.2.4. Sensitivity Analysis of Detecting Foreign Body Noise Point

**Figure 5:**
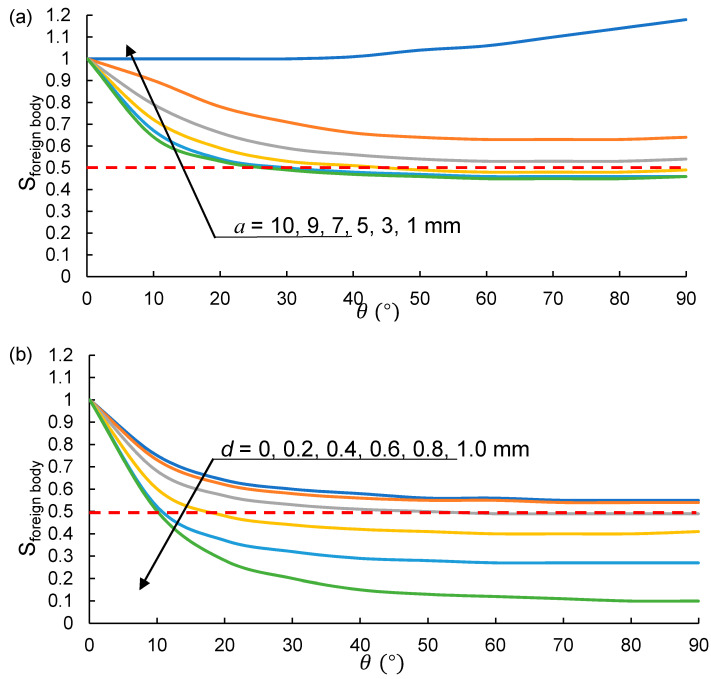
(**a**) The impact of θ and *a* on *S_foreign body_* when *d* = 0.5 mm and (**b**) the impact of θ and *d* on *S_foreign bod_* when *a* = 10 mm.

A point is detected to be a foreign body noise point if the calculated *S_foreign body_* < 0.5. Figure 5 shows the relationship between the *S_foreign body_* and the ellipsoid parameter a, θ and *d*. In Figure 5a, *d* is fixed as 0.5 mm, and *c* = 1 mm. It is found that *S_foreign body_* is always around 1 with the increase of θ (when *a* = *c* = 1 mm corresponding to a spherical detection). It is concluded that the spherical detection cannot denoise the foreign body points, because *S_foreign body_* < 0.5 is required to detect a point as a foreign body noise point.

On the other hand, *S_foreign body_* decreases with the increase of θ and *a* (when a > *c* = 1 mm). However, the increases of a shows the limited effect on *S_foreign body_*. The *S_foreign body_* begins to drop below 0.5 until that a is greater than 9 mm. Therefore, *a* is fixed as 10 mm in Figure 5b for the purpose of investigating the effect of *d* on *S_foreign body_*. From Figure 5b, it is found that *S_foreign body_* is less than 0.5 when the ellipsoid parameter a is taken as 10 mm with *d* > 0.6 mm and θ > 20°, which has a good distinction between foreign body noise points and ground points.

Moreover, *c* (the ellipsoid polar radius) equals to 1 mm in the assumption to simplify the calculation, and the distance from the foreign body point to the pavement plane is actually from 0 to 0.60*c*. By reduce the value of *c*, the accepted distance from the pavement plane can be controlled to a relatively small value to eliminate more foreign body noise points and ground points far from the pavement.

### 4.3. Sensitivity Analysis of the Ellipsoid Detection

Table 1 illustrates the normalized total intersection area and the sensitivity to the influencing factors for the three types of points. For the ground point, the total intersection area in its ellipsoid neighborhood remains unchanged. For the scattered noise point, the total intersection area in its ellipsoid neighborhood is less than half of that of the ground points when its distance from the pavement plane (*d*) is greater than 0.71*c*, and it is identified a noise point and to be eliminated. For the foreign body noise point, the total intersection area in its ellipsoid neighborhood is less than half of that of the ground points under the conditions of *a* equals to 10*c*, the angle between the foreign body and the pavement is less than 160° ($\theta >20^{{}^{\circ}}$), and its distance from the pavement plane (*d*) is greater than 0.6*c*.

## 5. Algorithm Verification

### 5.1. Verification by Simulated Point Cloud

**Figure 6:**
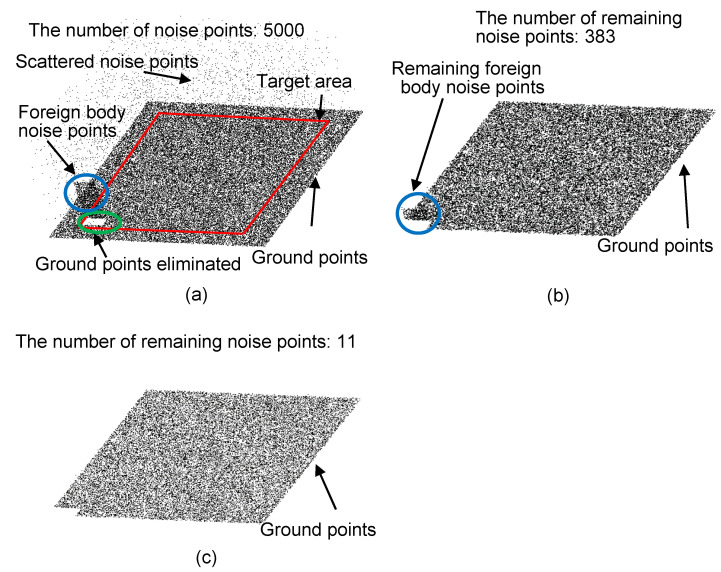
(**a**) The simulated point cloud before denoising, (**b**) denoised result by spherical detection (relevant factors refer to No. 4 in Table 2) and (**c**) denoised result by ellipsoid detection (relevant factors refer to No. 3 in Table 2).

To verify the validity of the proposed ellipsoid detection, the detection algorithms are compared in terms of the number of noise points eliminated based on a simulated point cloud by MATLAB. A laptop with a CPU of intel i5 7200U (2.5 GHz) and RAM of 16 G (1866 MHz). The simulated point cloud includes the ground points, the scattered points, and the foreign body points, which is normally seen during the pavement scanning (Figure 6a). 

Figure 6b,c are the denoised results by utilizing the spherical neighborhood and the ellipsoid neighborhood, respectively. It can be seen that foreign body points in the left lower corner of Figure 6b have not been identified as noise point and eliminated, which means that the spherical detection cannot effectively eliminate the foreign body noise points. From Figure 6c, the ellipsoid detection can effectively eliminate both the scattered noise points and foreign body noise points. Particularly, there are 383 noise points left in Figure 6b, and there are only 11 noise points left in Figure 6c, which suggests that the ellipsoid detection has a better denoising result than the spherical detection. 

The detailed denoising results by the two detection algorithms and the influences of parameters (*a*, *c, h_c_*, and $n_{c}$) are listed in Table 2. It can be seen that the ellipsoid detection can eliminate more scattered noise points and foreign body noise points than the spherical detection. Particularly, for the ellipsoid detection, when $n_{c}$ equals to 3, at most 31 noise points (in No. 2) are remained after denoising process. With the decrease of $n_{c}$, the denoising critical value determined by Equation (1) will increase, which makes more ground points eliminated. Therefore, when $n_{c}$ decreases from 3 (No. 1 in Table 2) to 2 (No. 3 in Table 2), the number of remaining noise points decreases by 1, but the number of remaining ground points decreases by 434. This means that $n_{c}$ = 3 is enough to eliminate noise points. 

As illustrated in Section 3.1, the smaller the ellipsoid, the lower the computing time. Comparing No. 1 and No. 2 Table 2, when the ellipsoid size reduces to the half of its origin one, the computing time changes from 21 s to 10 s. However, the number of remaining noise points increases by 19, and the number of remaining ground points decreases by 434. It means that the ellipsoid size cannot be reduced indefinitely when the ratio of *c/a* is unchanged, which may mistakenly eliminate some ground points.

### 5.2. Verification by the Scanned Point Cloud

Due to the influence of traffic, load, and the pavement material types, the actual scanned pavement point cloud is more complex than the simulated point cloud. To verify the feasibility of the proposed algorithm to the real pavement point cloud, two types of scanned point clouds, namely, the flat concrete pavement and the concrete pavement with stones and kerbs are selected for denoising.

#### 5.2.1. Flat Concrete Pavement

**Figure 7:**
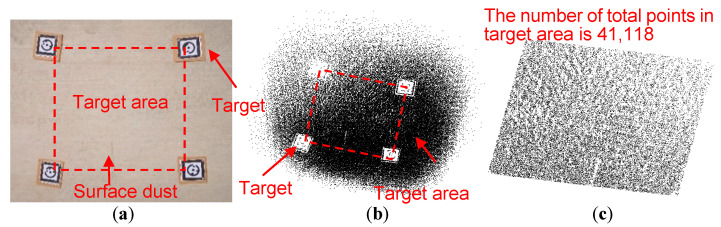
(**a**) The photo of the flat concrete pavement, (**b**) the original point cloud and (**c**) the original point cloud within in the target area.

The flat concrete pavement and its original scanned point cloud are shown in Figure 7a,b, respectively. The point cloud within the four targets is used for denoising (Figure 7c). The original point cloud consists of 41,118 points within the target area, the main noise points are the scattered noise points. 

As shown in Table 3, different denoising parameters (*a, c, h_c_*, *n_c_*, and *N_c_*) are used to denoise the point cloud, and the numbers of eliminated points are 263, 748, 670, 1140, 40, and 382 for No. 1–No. 6 parameter values in Table 3. Particularly, *a* is fixed as 0.01 m and the shape of the ellipsoid is changed by changing *c*. $N_{c}$ is fixed as 3 because this value is appropriate to eliminate noise points by adjusting *c*, *h_c_*, and *n_c_*. 

For ellipsoid detection, with the decrease of *c/a*, *S_foreign body_* (determined by Equation (14)) will decrease (shown in Figure 6), which makes more foreign body points eliminated. Moreover, with the decrease of $n_{c}$, the denoising critical value determined by Equation (1) will increase, which makes more potential noise points eliminated. Particularly, No.4 denoising parameter combination (*a* = 0.01, *c* = 0.001, *h_c_* = 6, *n_c_* = 2, and *N_c_* = 3) eliminates the most noise points and still well preserves the ground points. For the spherical detection, the number of eliminated points increases with the decrease of *n_c_*, similar to the ellipsoid detection.

#### 5.2.2. Concrete Pavement with Stones and Kerbs

**Figure 8:**
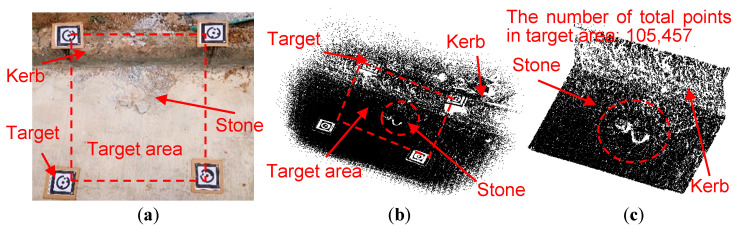
(**a**) The photo of the flat concrete pavement with stones and kerbs, (**b**) the original point cloud and (**c**) the original point cloud within the target area.

The actual concrete pavement with stones and kerbs and its original scanned point cloud are shown in Figure 8a,b, respectively. The point cloud within four targets is used for the denoising (Figure 8c), which consists of 105,457 points.

**Figure 9:**
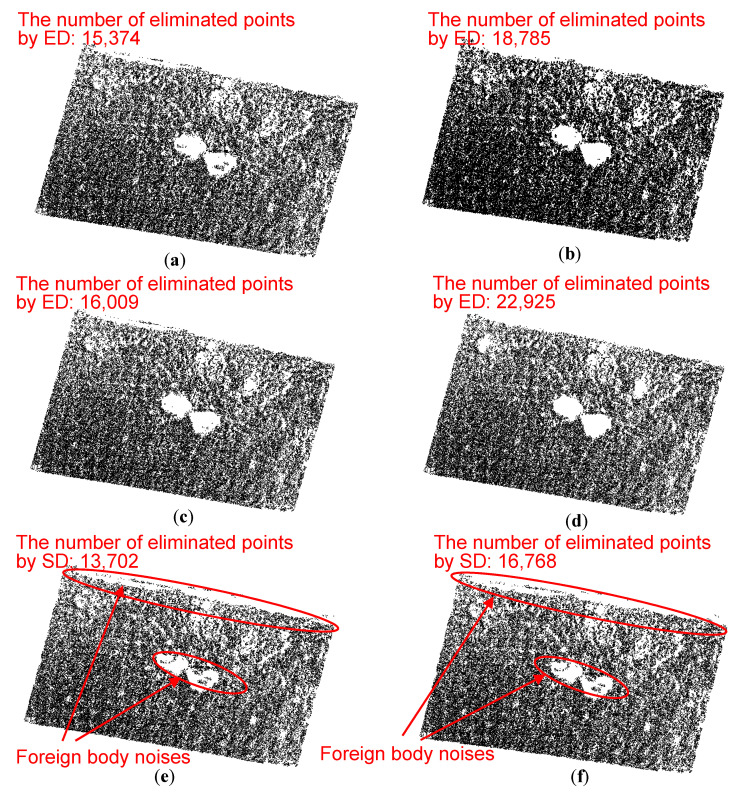
The denoising results of the flat concrete pavement by using (**a**) No. 1, (**b**) No. 2, (**c**) No. 3, (**d**) No. 4, (**e**) No. 5 and (**f**) No. 6 parameter values in Table 4, respectively. (ED: ellipsoid detection; SD: spherical detection).

As shown in Table 4, different denoising parameters (*a*, *c*, *h_c_*, *n_c_*, and *N_c_*) are used to denoise the point cloud, and the results are shown in Figure 9. Particularly, *a* is fixed as 0.01 m and the shape of the ellipsoid is changed by changing *c*. $N_{c}$ is fixed as 3, because this value is appropriate to eliminate noise points by adjusting *c*, *h_c_*, and *n_c_*. 

For the ellipsoid detection, with the decrease of $n_{c}$, the denoising critical value determined by Equation (1) will increase, which makes more potential noise points eliminated. According to Figure 9a–d, No. 4 denoising parameter combination (*a* = 0.01, *c* = 0.001, *h_c_* = 6, *n_c_* = 1.5, and *N_c_* = 3) eliminates most noise points and still well preserve the ground points. 

For the spherical detection, the number of eliminated points increases with the decrease of *n_c_*, similar to the ellipsoid detection. In the denoised results by the spherical detection (Figure 9e,f), the foreign body noise points with high *z*-coordinates are eliminated, while the foreign body noise points close to the pavement are remained. Because the spherical detection homogenizes the contribution of *x*-, *y*-, and *z*-coordinates of point cloud, and cannot eliminate foreign body (such as stones or kerbs) noise points close to the pavement.

It is concluded that the cell meshing ellipsoid detection can eliminate more scattered noise points and foreign body noise points than the spherical neighborhood detection. Particularly, with the decrease of *c*/*a* or *n_c_*, more points will be eliminated, which may eliminate more potential noise points and the ground points far from the pavement plane.

## 6. Conclusions

The conventional 3D point cloud denoising algorithm emphasizes on improving the smoothness of the point cloud, however, ignores the horizontal characteristics of the pavement, which makes it difficult to eliminate the foreign body noise points and the scattered noise points close to the pavement plane. Meanwhile, the conventional denoising algorithm is lack of the cell meshing process and demands for calculating the distance between any two points, making the computing time very long. In this study, a new denoising algorithm based on the cell meshing ellipsoid detection is proposed, which reduces the computing time and highlights the contribution of horizontal coordinates to the critical value of denoising. To confirm the accuracy and efficiency of the proposed algorithm, the ellipsoid detection difference between the ground points, the scattered noise points and the foreign body noise points is theoretically analyzed. The ellipsoid detection algorithm is then quantitatively verified by the simulated point cloud and the practically scanned pavement point cloud, and the comparison is finally conducted between the ellipsoid neighborhood detection and the traditional spherical neighborhood detection. 

It is found that the denoising algorithm based on the cell meshing ellipsoid detection enlarges the contribution of the horizontal coordinates for distinguishing noise points. *c*/*a*, *h_c_, n_c_,* and *N_c_* are the denoising parameters, in which $n_{c}$ and *N_c_* are used to control the denoising degree determined by Equations (1) and (2). By reducing the denoising parameters (*c*/*a*, *h_c_, n_c_,* and *N_c_*), more foreign body noise points and ground points may be eliminated, because the critical value is increased. Compared to the traditional spheroid neighborhood detection, the ellipsoid detection can better eliminate the scattered noise points close to the pavement plane and the foreign body noise points. At the same time, the cell meshing procedure reduces the computing time by reducing the spatial scale of the ellipsoid detection.

The proposed algorithm is an improvement based on the spherical neighborhood detection of the ordinary object denoising algorithm. It can quickly and efficiently eliminate the scattered noise points. In addition, it has a good denoising effect on the foreign body noise points such as the stones and the kerbs. This algorithm can provide a practical, accurate and efficient way of processing point cloud for the later 3D reconstruction of pavement and defect identification.

## Figures and Tables

**Table 1:** The intersection area in the ellipsoid neighborhood of different types of points.

Point Types	Intersection Area after Normalization	Sensitivity	Explanation
Ground point	1	constant	The numbers of points in a ground point neighborhood are almost the same.
Scattered noise point	As calculated by Equation (8)	*S_scatter_* < 0.5 when *d* > 0.71*c*	If the distance between a scattered noise point and the road plane is larger than 0.71*c*, the number of points in its neighborhood will be less than half of that in a ground point neighborhood.
Foreign body noise points	As calculated by Equations (12)–(14)	*S_foreign body_* < 0.5 when *a* = 10*c*, *d* > 0.6*c* and *θ* > 20°	Under the conditions of *a* equals to 10*c*, the distance between a foreign body noise point and the road plane is larger than 0.6*c*, and the angle between the foreign body and the road plane is larger than 20°, the number of points in its neighborhood will be less than half of that in a ground point neighborhood.

**Table 2:** The denoising results of the simulated point cloud by different denoising parameters.

		No.	*a* (m)	*c* (m)	*h_c_*	*n_c_*	*N_c_*	Number of Remaining Ground Points	Number of Remaining Scattered Noise Points	Number of Remaining Foreign Body Noise Points	**Computation Time (s)**
**Before denoising**		-	-	-		-	-	31,786	4000	1000	-
**After denoising**	**Ellipsoid** **detection**	1	0.02	0.002	3	3	3	31,463	1	11	21
		2	0.01	0.001	6	3	3	28,477	2	29	10
		3	0.02	0.002	1	2	3	31,029	1	10	20
	**Spherical** **detection**	4	0.02	0.02	1	3	3	31,778	89	294	25

**Table 3:** The denoising results of the concrete pavement point cloud without foreign body noise points by different denoising parameters.

		No.	*a* (m)	*c* (m)	*h_c_*	*n*_c_	*N_c_*	Number of Eliminated Points	Number of Preserved Points
**Before denoising**		-	-	-		-	-	0	41,118
**After** **denoising**	**Ellipsoid** **detection**	1	0.01	0.002	3	3	3	263	40,855
		2	0.01	0.002	3	2	3	748	40,370
		3	0.01	0.001	6	3	3	670	40,448
		4	0.01	0.001	6	2	3	1140	39,978
	**Spherical** **detection**	5	0.01	0.01	1	3	3	40	41,078
		6	0.01	0.01	1	2	3	382	40,736

**Table 4:** The denoising results of the concrete pavement point cloud with stone and kerb noise points by different denoising parameters.

		No.	*a* (m)	*c* (m)	*h_c_*	*n*_c_	*N_c_*	Number of Eliminated Points	Number of Preserved Points
**Before denoising**		-	-	-		-	-	0	105,457
**After** **denoising**	**Ellipsoid** **detection**	1	0.01	0.002	3	3	15,374	90,083	40,855
		2	0.01	0.002	3	1.5	18,785	86,672	40,370
		3	0.01	0.001	6	3	16,009	89,448	40,448
		4	0.01	0.001	6	1.5	22,925	82,532	39,978
	**Spherical** **detection**	5	0.01	0.01	1	3	13,702	91,755	41,078
		6	0.01	0.01	1	1.5	16,768	88,689	40,736

## Data Availability

Data is contained within the article.
